# Accumulation of γδ T cells in visceral fat with aging promotes chronic inflammation

**DOI:** 10.1007/s11357-022-00572-w

**Published:** 2022-04-28

**Authors:** Maria E. C. Bruno, Sujata Mukherjee, Whitney L. Powell, Stephanie F. Mori, Franklyn K. Wallace, Beverly K. Balasuriya, Leon C. Su, Arnold J. Stromberg, Donald A. Cohen, Marlene E. Starr

**Affiliations:** 1 Department of Surgery, University of Kentucky, 760 Press Ave, HKRB 238, Lexington, KY 40536 USA; 2 Department of Pharmacology and Nutritional Sciences, University of Kentucky, Lexington, KY 40536 USA; 3 Department of Statistics, University of Kentucky, Lexington, KY 40536 USA; 4 Department of Microbiology, Immunology, and Molecular Genetics, University of Kentucky, Lexington, KY 40536 USA

**Keywords:** Adipose tissue, Aging, Obesity, Chronic inflammation, Gamma delta T cells

## Abstract

Adipose tissue dysfunction is strongly linked to the development of chronic inflammation and cardiometabolic disorders in aging. While much attention has been given to the role of resident adipose tissue immune cells in the disruption of homeostasis in obesity, age-specific effects remain understudied. Here, we identified and characterized a population of γδ T cells, which show unique age-dependent accumulation in the visceral adipose tissue (VAT) of both mice and humans. Diet-induced obesity likewise increased γδ T cell numbers; however, the effect was greater in the aged where the increase was independent of fat mass. γδ T cells in VAT express a tissue-resident memory T cell phenotype (CD44^hi^CD62L^low^CD69^+^) and are predominantly IL-17A-producing cells. Transcriptome analyses of immunomagnetically purified γδ T cells identified significant age-associated differences in expression of genes related to inflammation, immune cell composition, and adipocyte differentiation, suggesting age-dependent qualitative changes in addition to the quantitative increase. Genetic deficiency of γδ T cells in old age improved the metabolic phenotype, characterized by increased respiratory exchange ratio, and lowered levels of IL-6 both systemically and locally in VAT. Decreased IL-6 was predominantly due to reduced production by non-immune stromal cells, primarily preadipocytes, and adipose-derived stem cells. Collectively, these findings suggest that an age-dependent increase of tissue-resident γδ T cells in VAT contributes to local and systemic chronic inflammation and metabolic dysfunction in aging.

**Supplementary Information:**

The online version contains supplementary material available at 10.1007/s11357-022-00572-w.

## Introduction

Adipose tissues are active endocrine organs, secreting an array of inflammatory mediators and hormones which have physiological effects on multiple organ systems [1–3]. Adipose tissue dysfunction (deleterious changes in cellularity, secretory profile, inflammatory state, and insulin responsiveness) is linked to metabolic derangements and chronic low-grade systemic inflammation, which are predictors of impaired physical function and frailty in the aged [4–7]. In fact, aging poses the greatest risk for the development of chronic conditions, such as hypertension, diabetes, and cardiovascular disease [7–11]. These conditions occur much more frequently in people as they get older [12], yet are more often studied in the context of obesity. While medical complications resulting from aging or obesity can be similar, recent research has demonstrated clear mechanistic differences related to altered adipose tissue function and underlying chronic inflammation in these states [6, 13–15]. Nevertheless, a majority of our current knowledge related to adipose tissue dysfunction has been derived from studies on obese animals and humans, and extrapolated to similar pathophysiologies in aging [2]. To understand age-associated chronic diseases, in the presence or absence of obesity, age-specific changes in adipose tissue physiology and function need to be more completely understood.

γδ T cells are a unique and poorly understood class of lymphocytes generally regarded for their role in barrier protection with functionally distinct subpopulations residing in epithelial tissues, including those of the skin, gut, and lung. In addition to responding to antigen presentation via the T cell receptor, similar to conventional T cells (regulatory, helper, and cytotoxic T cells of the αβ lineage) of the adaptive immune system, γδ T cells can respond directly to cytokines and other intact proteins without antigen processing and presentation, and have the capacity to phagocytize much like innate immune cells [16–18]. γδ T cell numbers are significantly reduced in the circulation of older people likely contributing to the suppressed immune response in the elderly [19–22]. Very little, however, is known about the role γδ T cells play in adipose tissue, especially as it pertains to aging [23].

In adipose tissues, contrary to their traditional function in infection control, γδ T cells appear to play major roles in maintaining homeostasis with respect to inflammation and insulin sensitivity. Adipose tissue γδ T cells have been shown to increase in number in mouse models of diet-induced obesity, where they promote macrophage accumulation, inflammation, and insulin resistance [24–26]. More recently, they were reported to regulate adipose tissue regulatory T cell homeostasis and thermogenesis in adolescent and young-adult mice (1–7 months of age) [27]. It is not known whether the phenotypes and functions put forth in these studies carry forward into old age and influence age-associated inflammation and disrupted metabolic homeostasis.

In this study, we share our discovery that γδ T cells are uniquely increased in visceral adipose tissue (VAT) in old age, and provide evidence that their age-associated increase is linked with a poor metabolic and inflammatory phenotype. We further define age-related changes in phenotype and function of adipose resident γδ T cells.

## Materials and methods

### Animals and husbandry

Male and female C57BL/6 mice were obtained from The Jackson Laboratory (Stock 664) or the National Institute on Aging. T cell receptor delta chain knockout mice (TCRδ KO, B6.129P2-Tcrd^tm1Mom^/J, Stock 2120) were obtained from The Jackson Lab and bred in-house. Mice were euthanized as young-adults (4–7 months old) or at old age (19–24 months old); age is specified for each experiment in figure legends. Mice were housed in pressurized intraventilated cages and maintained in an environment under controlled temperature (21–23 °C), humidity (30–70%), and lighting (14 h/10 h, light/dark) with free access to drinking water and chow (Teklad Global No. 2918). A subset of mice was subjected to high-fat feeding (Teklad TD.88137 42% kcal from fat) alongside control diet-fed mice (Teklad TD.08485, 13% kcal from fat); diets were given ad libitum. All procedures were approved by the Institutional Animal Care and Use Committee at the University of Kentucky, and performed in accord with the National Institutes of Health guidelines for ethical animal treatment.

### Murine sample collection

Mice were deeply anesthetized by isoflurane inhalation, laparotomy performed, and blood collected from the inferior vena cava (IVC) by syringe needle with 10% volume of 0.1 M sodium citrate. Blood was immediately centrifuged (2500 × *g*, 4 °C, 15 min) to obtain plasma which was stored at − 80 °C. Subsequently, the IVC was cut, and the entire vasculature was perfused with 30 mL physiological saline through the cardiac ventricles to eliminate circulating cells. For protein or gene expression analyses, tissues were carefully dissected, flash frozen in liquid nitrogen, and stored at − 80 °C. For flow cytometry studies, fresh tissues (visceral gonadal fat pads (VAT), subcutaneous inguinal fad pads (SAT), spleen, and dorsal portion of skin) were dissected after perfusion and kept on ice until processing.

### Human sample acquisition

Fresh visceral adipose tissue specimens (mesenteric fat, perirenal fat, and omentum) were obtained from patients undergoing surgery at the University of Kentucky Medical Center. Sample collection was facilitated in a deidentified manner through the University of Kentucky Center for Clinical and Translational Science, and approved by the University of Kentucky Institutional Review Board (Study # 44734). Specimens were retrieved from the operating room and kept on ice until processing.

### Tissue processing for single-cell suspensions

#### *Adipose tissue*

VAT and SAT were dissected from mice, weighed, and minced with scissors. Minced tissues were transferred to ice cold digestion buffer (0.5% BSA in HBSS with Ca^2+^Mg^2+^) with collagenase (1 mg/mL) and incubated on a tube rocker at 37 °C for 50 min with vigorous shaking by hand every 10 min. Prior to the final 10 min, 10 mM EDTA was added. Digested cells were passed through a 200-μm strainer and centrifuged (500 × g, 10 min, 4 °C) to separate mature adipocytes from stromal vascular fraction (SVF) cells. The top adipocyte layer was aspirated and discarded. The cell pellet containing SVF cells was treated with 3 mL red blood cell (RBC) lysis buffer for 5 min, passed through a 70-μm strainer and centrifugation repeated. SVF cells were resuspended in an appropriate volume of digestion buffer for subsequent processing/analysis. Human VAT was processed in similar fashion.

#### *Blood*

RBC lysis buffer (20-mL) was added to whole blood (approx. 1-mL per mouse) in a 50-mL conical tube. Cell suspensions were incubated on ice for 5 min with occasional shaking, passed through a 70-μm strainer, washed, and centrifuged. RBC lysis was repeated, followed by another round of centrifugation. White blood cells (WBC) were resuspended in an appropriate volume of digestion buffer for subsequent processing/analysis.

#### *Spleen*

Spleen was mashed with a syringe plunger through a 100-μm cell strainer, washed with 20-mL digestion buffer and centrifuged at 500 × *g* for 10 min. RBC lysis was performed and cells were passed through a 70-μm strainer. After a second round of centrifugation, spleen cells were resuspended in an appropriate volume of digestion buffer.

#### *Skin*

A 3 × 3 cm area of skin was shaved on the dorsal aspect of the mouse, Nair was applied for 3 min, and the skin was washed thoroughly with running water. Subsequently, a 2 × 2 cm piece of skin was excised and scraped of excess adipose/connective tissue. Skin was minced in a 35-mm Petri dish containing 2 mL of digestion cocktail (300 µg/mL Liberase, 50 U/mL Dnase I in 5% FBS-RPMI) and incubated at 37 °C for 90 min with agitation every 30 min. Digested skin was then mashed through a 100-µm cell strainer and washed. Samples were centrifuged at 350 × *g* for 5 min, the supernatant aspirated, and the pellet treated with RBC lysis buffer. Cells were passed through a 70-µm cell strainer, another round of centrifugation performed, and the resulting pellet resuspended in an appropriate volume of digestion buffer.

### Flow cytometry

Single cell suspensions were stained with Fixable Viability Dye eFluor 450 (eBioscience 65-0843-14) according to the manufacturer’s protocol, and Fc receptor blocking was performed using TruStain FcX (Biolegend 156603) for 10 min on ice. Cells were further incubated for 30 min at 4 °C in the dark with respective antibodies for cell surface staining; antibodies used are described in Supplementary Table 1 and cell surface identifiers are described in Supplementary Table 2. Stained cells were fixed with 4% paraformaldehyde (Biolegend 420801) for 20 min. For human samples, the same procedure was performed with Human TruStain FcX™ (BioLegend 422302), and antibodies as noted in Supplementary Table 1. For intracellular staining, SVF cells were first incubated in vitro with GolgiPlug protein transport inhibitor (BD 555029) in 5% FBS-RPMI for 4 h at 5% CO_2_, 37 °C. Following cell surface staining and fixation, cells were permeabilized according to standard protocol (BD 51-2091KZ) and intracellular staining was performed using antibodies for IFNγ, IL-17A, IL-6, and respective isotype controls (see Supplementary Table 1). For detection of IFNγ and IL-17A, cells were also treated with Cell Stimulation Cocktail (Invitrogen 00-4970-93) during protein transport inhibition. For intracellular staining of IL-6, cell stimulation was not performed. Murine and human cells were analyzed on a FACSymphony A3 Cell Analyzer (BD, San Jose, CA). Analysis of flow cytometry data was performed using the FlowJo data analysis software (FlowJo, LLC, Ashland, OR).

### Immunomagnetic selection of cell fractions

WBCs and VAT SVF cells were purified from 24-month old mice (3 pools of 8 aged mice each) and 4-month old mice (3 pools of 10 mice each) for sequential magnetic separation of γδ and αβ T cells. Pools of mice were used to achieve the required starting material for magnetic separation kits. *Selection of PE-labeled *γδ* T cells:* γδ T cells were purified by positive selection for γδ TCR with anti-mouse γδ TCR antibody (STEMCELL Technologies 60104PE) and EasySep mouse PE positive selection kit with magnetic particles (STEMCELL Technologies 17666), following the manufacturer’s directions. Magnetically isolated γδ TCR positive cells were washed 5 times to obtain pure cells. *Selection of FITC-labeled αβ T cells:* The initial supernatant, devoid of γδ T cells and containing the remaining cells, was used to positively select for CD3 positive cells (αβT cells) with anti-mouse CD3 antibody (STEMCELL Technologies 60015FI) and EasySep mouse FITC positive selection kit (STEMCELL Technologies 17668). Magnetically isolated CD3 positive cells were washed 5 times to obtain pure cells. Purity of the fractions was assessed by flow cytometry.

### nanoString nCounter analyses

Immunomagnetically purified γδ T cells and αβ T cells from WBCs and VAT SVF were disrupted in RNA lysis buffer by vigorous vortexing for 2 min. Total RNA was purified using the PureLink™ RNA mini kit according to manufacturer’s directions (Invitrogen 12183018A) and DNA was removed using the DNA-free™ kit (Invitrogen AM1906). The concentration of the RNA was determined by measuring absorbance at 260 nm using a NanoDrop One (ThermoFisher Scientific), and integrity was confirmed through visualization of 18S and 28S RNA bands using an Agilent 2100 Bioanalyzer. Quantification of mRNA transcripts was performed by nanoString nCounter hybridization using at least 10–50 ng purified RNA and the pre-designed mouse PanCancer Immune Profiling Panel, which includes 770 immune profiling genes and 40 internal control genes (NanoString Technologies, Seattle, WA).

### Quantitative real-time RT-PCR

RNA was extracted from frozen tissues as previously described [28]. For adipose tissues, prior to the addition of chloroform, the homogenate was centrifuged at 10,000 rpm for 10 min and the upper lipid layer aspirated. Equivalent amounts of RNA were reverse transcribed into cDNA using SuperScript III First-Strand Synthesis SuperMix (Invitrogen 11752–050), according to the manufacturer’s protocol. TaqMan assays were purchased from ThermoFisher Scientific and qRT-PCR was performed on a QuantStudio 3 Real-Time PCR machine (Applied Biosystems). Target gene expression was normalized to hypoxanthine–guanine phosphoribosyl transferase (HPRT) expression as an endogenous control, and fold change was calculated as 2^−(ΔΔCT)^, using the mean ΔCT of the control group as a calibrator. TaqMan assays used were Mm00446190_m1 and Mm03024075_m1 for IL-6 and HPRT, respectively.

### *In vivo *metabolic phenotype analysis

Metabolic analyses were carried out according to rigorous SOPs established and validated within the Energy Balance and Body Composition Core Facility at the University of Kentucky Center of Research in Obesity and Cardiovascular Disease. Body composition was determined by Echo-MRI (Echo Medical Systems, Houston, TX, USA) and oxygen consumption (VO_2_), respiratory exchange ratios (RER), activity levels, and food consumption were monitored for at least 3 consecutive days and nights after 1 week for acclimation, before data recording using the TSE-LabMaster Indirect Calorimetry Research Platform (Chesterfield, MO).

### Statistical analyses

Comparisons of continuous variables were analyzed first using overall two-way ANOVA, Hotelling’s T^2^ tests, or Student’s *t*-test. Multiple comparisons of continuous variables were then performed using Fisher’s Least Significant Difference. Correlation between continuous variables was analyzed using Pearson’s Correlation Coefficient. All analyses were done using R (R Core Team, Vienna, Austria).

## Results

### γδ* T cells are increased by aging specifically in visceral adipose tissue*

**Fig. 1:**
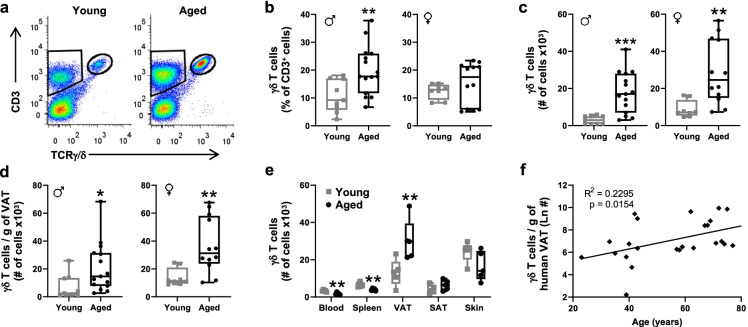
γδ T cells are increased by aging specifically in visceral adipose tissue. **a** Representative flow cytometry plots of γδ T cells in visceral adipose tissue. **b** Percentage, **c** Total number, and **d** Number per gram of adipose tissue of γδ T cells was quantified in young (4–6 months) and aged (19–25 months) male (*n* = 9 young, *n* = 15 aged) and female (*n* = 8 young, *n* = 12 aged) C57BL/6 mice. Statistical differences were determined by two-way ANOVA with Fisher’s Least Significant Difference for multiple comparisons. **e** Number of γδ T cells in blood and tissues of young and aged mice (*n* = 5, each; 6 and 19 months old, respectively). Statistical differences were determined by a two-sample Hotelling’s T^2^ test followed by Student’s *t*-test. Data are expressed in box plots from minimum to maximum values with a bar representing the mean; each symbol represents an individual mouse. *p < 0.05; **p < 0.01; ***p < 0.001. **f** Number of γδ T cells per gram of human visceral adipose tissue according to age; each symbol represents a sample from an individual subject. Statistical significance of the correlation was assessed by Pearson correlation coefficient. VAT, visceral adipose tissue; SAT, subcutaneous adipose tissue

We previously reported that expression of genes within the T-Cell-Receptor Pathway are significantly over-represented in gonadal visceral adipose tissue of aged (24 months old) compared to young (4 months old) male mice. Among genes within this pathway, the T cell receptor gamma constant region (TCRγ-C), a gene expressed exclusively by γδ T cells, showed a significant sixfold increase by aging [29]. Here, to evaluate whether this age-associated increase in gene expression corresponds to an increase in γδ T cell number with age, we surveyed γδ T cells in the stromal vascular fraction (SVF) of gonadal visceral adipose tissue (VAT) by flow cytometry (Fig. 1a, Representative plots, full gating scheme shown in Supplementary Fig. 1). Male mice showed a significant increase in percentage of γδ T cells by aging. Female mice showed a similar trend, though not reaching significance (Fig. 1b). Both males and females displayed a significant increase in total number of γδ T cells in VAT (Fig. 1c), which remained significant after adjusting for fat mass (Fig. 1d), suggesting that the increase is independent of adiposity. Inversely, the percentage of conventional T cells (T_conv_) decreased in the aged VAT, but total numbers were moderately increased by aging without a significant increase after adjusting for fat mass (Supplementary Fig. 2). The age-associated increase in γδ T cells was unique to VAT, not being observed in blood, spleen, subcutaneous adipose tissue (SAT), or skin (Fig. 1e). Importantly, the increase in γδ T cells with aging was also observed in human VAT (Fig. 1f).

### *Age-associated accumulation of VAT *γδ* T cells is independent of adiposity, yet further augmented by long-term high fat feeding*

**Fig. 2:**
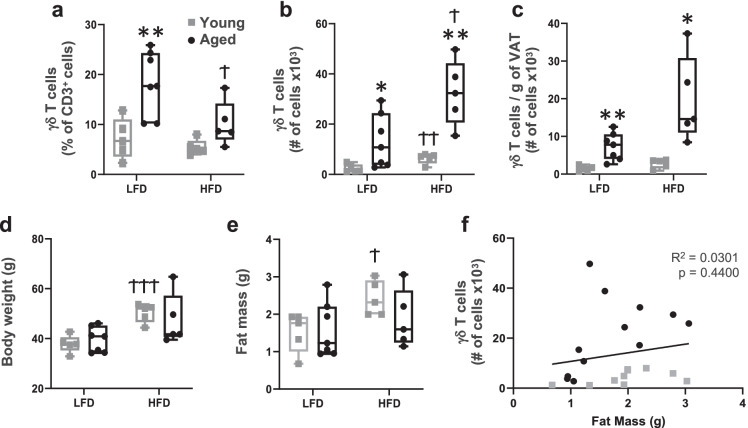
Age-associated increase of visceral adipose tissue γδ T cells is independent of adiposity, yet further augmented by long-term high fat feeding. Young (7 months at study endpoint) and aged (23 months at study endpoint) male C57BL/6 mice were fed a low-fat diet (LFD) or high-fat diet (HFD); young mice were fed for 20 weeks and aged mice were fed for 12 months to mimic an obese life-style (*n* = 5–7 per group). **a** Percentage of total T cells, **b** Total number, and **c** Number per gram of adipose tissue of γδ T cells were quantified in visceral adipose tissue by flow cytometry. **d** Body weight and **e** Fat mass were assessed at the study endpoint. Data are expressed in box plots from minimum to maximum values with a bar representing the mean; each symbol represents an individual mouse. Statistical differences were determined by two-way ANOVA with Fisher’s Least Significance Difference for multiple comparisons. *indicates significance between young and aged within the same diet group. †indicates significance between LFD and HFD within the same age group. * or †*p* < 0.05; ** or ††*p* < 0.01; *** or †††*p* < 0.001. **f** Correlation of total γδ T cell number with grams of VAT. Statistical significance of the correlation was assessed by Pearson correlation coefficient

It was previously reported that γδ T cells were increased in number in VAT of young mice fed various high-fat diets for periods ranging from 5 to 24 weeks [24, 26]. To further evaluate the effect of obesity on VAT γδ T cells in aging, we subjected mice to long-term high-fat feeding for 12 months (beginning at 11 months of age and terminating at 23 months of age). Young mice given the same diets were fed for 20 weeks (beginning at 2 months of age and terminating at 7 months of age) to mimic standard diet-induced obesity models. Similar to previous reports, HFD increased total number, but not percentage of γδ T cells in young mice (Fig. 2a and b, gray squares), and the increase was proportional to the increase in adiposity, assessed by adjusting for fat mass (Fig. 2c). In aged mice, the average percentage of γδ T cells was decreased by HFD (Fig. 2a, black circles), but total number of cells was significantly increased (Fig. 2b). Adjusting for fat mass in aged mice did not abrogate the significant increase in γδ T cell number (Fig. 2c), indicating that aging with high-fat feeding may have an additive effect with respect to γδ T cell accumulation in VAT. Of note, while body weight (Fig. 2d) and fat mass (Fig. 2e) increased expectedly by short-term HFD in young mice, long-term HFD in the aged did not further increase body weight or fat mass. Moreover, despite young HFD-fed mice having fat mass similar to aged HFD-fed mice (Fig. 2e), γδ T cell number was higher in both LFD- and HFD-fed aged mice (Fig. 2c), and overall no correlation between fat mass and number of γδ T cells was observed (Fig. 2f).

### γδ* T cells in VAT display a tissue-resident memory (TRM) phenotype and predominantly express IL-17A*

**Fig. 3:**
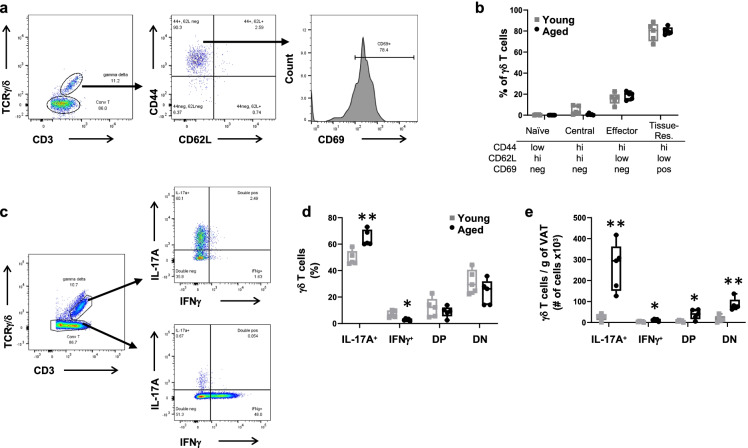
γδ T cells in visceral adipose tissue display a tissue-resident memory (T_RM_) phenotype and predominantly express IL-17A. γδ T cells from visceral adipose tissue of young (7 months) and aged (23 months) mice were assessed for memory phenotype by flow cytometry using the cell surface markers CD44, CD62L, and CD69; **a** Gating scheme, and **b** Proportion of cells within each phenotype as a percent of total γδ T cells. γδ T cells from visceral adipose tissue of young (7 months) and aged (23–24 months) mice were assessed for intracellular IL-17A and IFNγ expression; **c** Gating scheme, **d** Percentage of total γδ T cells, and **e** Number of γδ T cell per gram of adipose tissue for each intracellular stain. Data are expressed in box plots from minimum to maximum values with a bar representing the mean; each symbol represents an individual mouse. Statistical differences were determined by Student’s *t*-test. *p < 0.05; **p < 0.01. DP, double positive; DN, double negative

To profile γδ T cells in VAT, we assessed memory phenotype by expression of standard cell surface markers CD44, CD62L, and CD69 [30–32]. In young and aged mice alike, VAT γδ T cells were predominantly tissue-resident memory (T_RM_) cells (CD44^hi^, CD62L^low^ and CD69^+^) with more than 80% of the population expressing the T_RM_ phenotype (Fig. 3a and b). Furthermore, γδ T cells were categorized into subtypes based on IL-17A or IFNγ production, which distinguishes their functional programming. Intracellular staining for these two cytokines showed that IL-17A^+^ γδ T cells made up the largest subset. Approximately 50% of total γδ T cells in young VAT and 65% of total γδ T cells in aged VAT were single-positive for IL-17A (Fig. 3c and d). Less than 10% of γδ T cells in both age groups were single-positive for IFNγ. In addition to these mutually exclusive populations, a subset of γδ T cells was positive or negative for both markers (double positive (DP) and double negative (DN), respectively). Total cell numbers per gram of VAT showed an age-associated increase for all γδ T cell subsets, although the IL-17A^+^ subset was clearly the most prevalent and showed a profound age-associated increase (Fig. 3e). In contrast, T_conv_ cells in VAT showed a preference for IFNγ or lacked both markers, with little age-related variance (Supplementary Fig. 3). In the spleen, γδ T cells were equally distributed between IL-17A^+^, IFNγ^+^, and DN subsets, while splenic T_conv_ cells were largely IFNγ^+^ or DN, with little variance by age (Supplementary Fig. 4).

### *Differential gene expression between young and aged VAT *γδ* T cells*

**Fig. 4:**
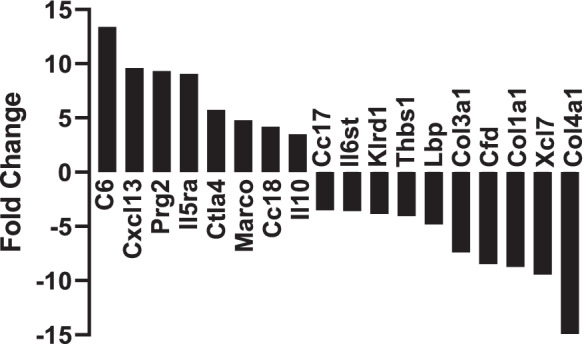
Age-associated changes in gene expression of visceral fat resident γδ T cells. γδ T cells were immunomagnetically purified from SVF of visceral adipose tissues obtained from young (4 months) and aged (24 months) mice using anti-TCRγ/δ antibody. RNA was isolated and transcriptome analyzed using nanoString nCounter Immune Profiling Panel. Expression of genes with significant alteration by aging, expressed as average fold change in aged compared to young, only those with fold change > 2.0 and *p* < 0.01 were included. SVF, stromal vascular fraction

To evaluate functional differences in young versus aged VAT γδ T cells with respect to inflammation, we performed transcriptome analyses on γδ T cells purified from VAT of young and aged mice, using the nanoString Immune Profiling Panel, which contains 770 genes related to inflammation and the immune response. The γδ T cell fractions, obtained by immunomagnetic separation, were 98% pure as assessed by flow cytometry (Supplementary Fig. 5, middle panel). Eight genes were significantly upregulated and ten genes were significantly downregulated by aging in VAT γδ T cells (Fig. 4, *p* < 0.01 and fold change > 2.0). These genes and their respective known functions are listed in Table 1. This analysis points to qualitative/functional changes in VAT γδ T cells with aging, in addition to the quantitative changes.

**Table 1:** Genes differentially expressed by age in visceral fat-resident γδ T cells

Gene symbol	Gene name	Function	Log 2 fold change	Linear fold change	p value
Upregulated in aged γδ T cells					
C6	Complement component 6	Complement system, phagocyte stimulation, bacterial pathogen clearance, NLRP3 inflammasome activation	3.7	13.4	0.0031
Cxcl13	Chemokine (CXC) ligand 13	T and B cell regulation and chemoattractant, inflammatory, infectious, and lymphoproliferative diseases	3.3	9.6	0.0008
Prg2	Proteoglycan 2	Immune hypersensitivity reactions, neutrophil and macrophage activation	3.2	9.3	0.0067
IL5ra	Interleukin 5 receptor, alpha	Eosinophil accumulation, biomarker and pharmacogenetic factor in asthma, Treg proliferation	3.2	9.1	0.0044
Ctla4	Cytotoxic T-lymphocyte-associated protein 4	Naïve T-cell activation, T cell motility and homeostasis	2.5	5.7	0.0081
Marco	Macrophage receptor with collagenous structure	Pattern recognition receptor, phagocytosis, pathogen clearance, facilitates ATM lipid uptake	2.3	4.8	0.0098
Ccl8	Chemokine (C–C) ligand 8 (MCP-2)	Chemoattractant for multiple immune cells, promotes inflammation	2.1	4.2	0.0123
Il10	Interleukin 10	Anti-inflammatory cytokine, M2 polarization in adipose tissue, promotes insulin resistance, thermogenesis	1.8	3.5	0.0138
Downregulated in aged γδ T cells					
Col4a1	Collagen alpha-1(IV) chain	Angiogenesis, regulates HIF-1^α^ and VEGF, expression in AT positively correlates with insulin resistance	− 3.9	− 14.9	0.0029
Xcl1	Chemokine (C) ligand (lymphotactin)	Chemotaxis and activation of lymphocytes, adipose stem cell homing	− 3.2	− 9.4	0.0138
Col1a1	Collagen alpha-1 (I) chain	Component of type I collagen, make up most connective tissues	− 3.1	− 8.7	0.0095
Cfd	Complement factor D	Stimulates glucose transport, inhibits lipolysis, promotes adipocyte differentiation	− 3.1	− 8.5	0.0021
Col3a1	Collagen alpha-1 (III) chain	Component of type III collagen, platelet aggregation, blood clotting	− 2.9	− 7.4	0.0139
Lbp	Lipopolysaccharide binding protein	Acute phase immune reactions, presents LPS to immune cell surface	− 2.3	− 4.8	0.0039
Thbs1	Thrombospondin 1	Involved in endothelial cell adhesion and angiogenesis, enhances preadipocyte proliferation	− 2.0	− 4.0	0.0052
Klrd1	Killer cell lectin-like receptor subfamily D, member 1 (CD94)	Binding with HLA-E on target cells facilitates activation and expansion of NK and T cell subsets, overexpressed in γδT-cell lymphoma	− 1.9	− 3.8	0.0150
Il6st	Interleukin 6 signal transducer (glycoprotein 130)	Cytokine signaling, modulates pro- and anti-inflammatory pathways	− 1.8	− 3.6	0.0026
Ccl17	Chemokine (C–C) ligand 17	Induces T cell chemotaxis and activation, drives inflammation and pain through GM-CSF signaling	− 1.8	− 3.5	0.0069

### *VAT *γδ* T cells have a distinct transcriptome compared to circulating *γδ* T cells in the blood*

**Fig. 5:**
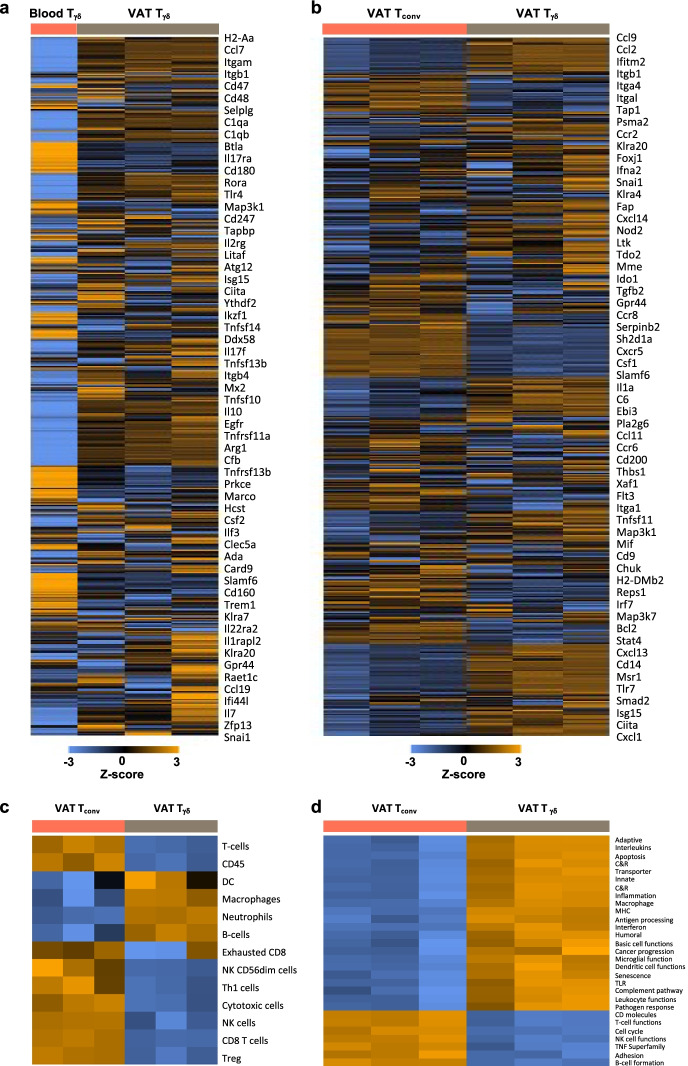
Visceral fat-resident γδ T cells have distinct transcriptomes compared to circulating γδ T cells and conventional T cells (T_conv_) in VAT. γδ T cells were immunomagnetically purified from whole blood and SVF of visceral adipose tissues obtained from aged mice (24 months) using anti-TCRγ/δ antibody and anti-CD3 antibody via sequential positive selection. RNA was isolated and transcriptome analyzed using nanoString nCounter Immune Profiling Panel. **a** Heatmap of the normalized gene expression data, from whole blood and VAT γδ T cells, scaled to give all genes equal variance, and generated via unsupervised clustering. **b** Heatmap of the normalized gene expression data, from VAT γδ T cells vs. VAT T_conv_ cells, scaled to give all genes equal variance, and generated via unsupervised clustering. **c** Heatmap showing abundance of genes normally related to specific cell types. **d** Heatmap of pathway scores. Orange indicates high expression or high z scores; blue indicates low expression or low z scores. Each sample represents cells pooled from 5–10 mice

To assess whether VAT γδ T cells differ functionally from circulating γδ T cells, we performed similar transcriptome analyses of immunomagnetically purified γδ T cells from VAT and blood of aged mice. A heatmap view of the normalized gene expression data shows a distinct transcriptome in blood versus VAT γδ T cells (Fig. 5a). These data suggest that the γδ T cells in VAT of aged mice are functionally distinct from peripheral γδ T cells circulating in the blood.

### *VAT *γδ* T cells and conventional T cells (T*_*conv*_*) have distinct transcriptomes*

To assess functional differences between γδ T cells and T_conv_ cells (i.e. αβ T cells) in VAT, we carried out the same transcriptome analyses on immunomagnetically purified T cells from aged mice, separated into γδ and αβ subgroups. Purity of the fractions was assessed by FACS and shown to be 98% and 96% pure, respectively (Supplementary Fig. 5). Principal component analysis demonstrated distinct clustering of gene expression profiles in γδ versus T_conv_ cells (Supplementary Fig. 6). A heatmap view of the normalized gene expression data likewise shows clear differences in overall transcriptomes (Fig. 5b). We next utilized the Immune Cell Profiling analyses of the nanoString platform to associate γδ T cells and T_conv_ cells with functional attributes. These analyses cluster genes into cell types based on the expression of genes characteristic of specific cell populations. While all the cells in our analyses were either γδ T cells or T_conv_ cells, the resulting heatmap allows us to draw assumptions regarding the function of each subset (Fig. 5c). For example, T_conv_ cells were enriched with genes normally attributed to CD8^+^ T cells, Th1 cells, cytotoxic cells, NK cells, and regulatory T cells. On the other hand, γδ T cells were enriched with genes normally attributed to macrophages, neutrophils, and B cells. These data suggest that VAT-resident γδ T cells are more innate in function compared to the adaptive immune phenotype of T_conv_ cells. Finally, pathway scores were assigned which further suggest a more inflammatory, innate, and myeloid-like function for VAT γδ T cells, while T_conv_ cells maintain a pathway profile typical of adaptive T lymphocytes (Fig. 5d). The most statistically significant differentially expressed genes between γδ T cells and conventional T cells are presented in Supplementary Table 3.

### *Improved metabolic phenotype and reduced inflammation in aged mice lacking *γδ* T cells (TCR*δ KO*)*

**Fig. 6:**
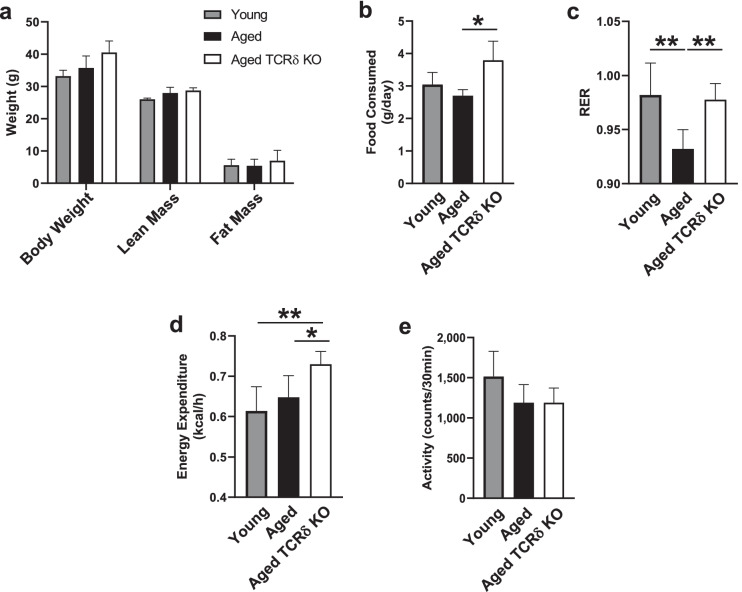
Improved metabolic phenotype in aged mice lacking γδ T cells (TCRδ KO). Young (4 months, *n* = 5) and aged (22 months, *n* = 5) C57BL/6 mice and aged (22 months, *n* = 4) TCRδ KO mice were subjected to measurements of **a** Body weight, **b** Food consumption, **c** Respiratory exchange ratio (RER), **d** Energy expenditure, and **e** Activity by indirect calorimetry experiments. Statistical differences were determined by one-way ANOVA with Fisher’s Least Significant Difference for multiple comparisons. *p < 0.05; **p < 0.01

To gain insight into the function of γδ T cells, we utilized TCRδ KO mice, which lack functional γδT cells due to disruption of the δ chain of the TCR which is required for their development [33]. Average body weight and composition were similar between aged TCRδ KO and wild-type mice, although TCRδ KO mice tended to be slightly heavier (Fig. 6a) and consumed more chow (Fig. 6b). Notably, respiratory exchange ratio (RER), often used to evaluate metabolic fitness [13, 34], was increased in aged mice lacking γδ T cells compared to age-matched wild-type mice (Fig. 6c), shifting the phenotype to match that of young mice. This is likely due to increased energy expenditure (Fig. 6d) despite similar activity levels (Fig. 6e).

**Fig. 7:**
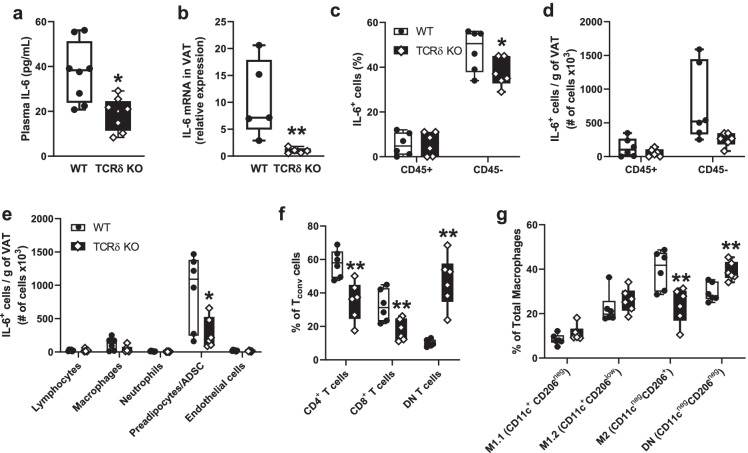
Reduced inflammation in aged mice lacking γδ T cells (TCRδ KO). Aged WT and aged TCRδ KO mice (24–27 months, *n* = 5–8) were euthanized for assessment of inflammatory cytokine IL-6. **a** IL-6 level in plasma was measured by ELISA and **b** IL-6 gene expression in VAT was measured by qRT-PCR. Intracellular staining of IL-6 in unstimulated cells was assessed by flow cytometry: **c** Percentage of IL-6^+^ cells and **d** Number of IL-6^+^ cells per gram of VAT compared between CD45^+^ immune cells and CD45 negative non-immune cells; **e** Number of IL-6^+^ cells per gram of VAT among major immune and non-immune cell subsets. **f** Percentage of T_conv_ cell subtypes and **g** Percentage of macrophage subtypes compared between WT and TCRδ KO mice. Statistical differences were assessed by Student’s *t*-test. *p < 0.05; **p < 0.01

Age-associated inflammation, based on IL-6 levels, was decreased systemically in the plasma (Fig. 7a), as well as locally in the VAT (Fig. 7b) of aged TCRδ KO mice compared to wild-type mice. Other tissues including kidney, lung, heart, and SAT did not show significant differences in IL-6, suggesting that reduced IL-6 in the KO mice is largely due to reduced IL-6 secretion by VAT (Supplementary Fig. 7). To determine which cells in the VAT are responsible for IL-6 production and whether loss of γδ T cells affects the cellular distribution of IL-6, we performed intracellular staining for IL-6 without stimulation (representative gating plots are shown in Supplementary Fig. 8). We identified that non-immune cells (CD45^neg^), as opposed to CD45^+^ immune cells, are the primary producers of IL-6 in the VAT (Fig. 7c). Total number of IL-6^+^ CD45^neg^ cells per gram of fat was significantly lower in TCRδ KO mice, suggesting that loss of γδ T cells reduces IL-6 production from non-immune stromal cells (Fig. 7d). This cellular compartment is primarily composed of committed preadipocytes, adipose-derived stem cells (ADSC), and endothelial cells [35–37]. Next, we compared IL-6 positivity among major immune (lymphocytes, macrophages, neutrophils) and non-immune (preadipocytes/ADSCs, endothelial cells) subsets by gating with commonly used cell surface markers (see gating strategy in Supplementary Table 2 and representative plots in Supplementary Fig. 8b–d). The CD45^neg^CD31^neg^ population, which excludes endothelial cells and consists of approximately 60% preadipocytes [36], was identified as the major subset of IL-6 producing cells. Additionally, the number of IL-6^+^ CD45^neg^CD31^neg^ (preadipocytes/ADSCs) was significantly reduced in TCRδ KO mice (Fig. 7e), suggesting that γδ T cells contribute to the inflammatory status of this cell subset. Unfortunately, we were unable to differentiate committed preadipocytes from ADSCs. Although immune cells are not responsible for the majority of IL-6 production in aged VAT, there is potential for their involvement in the signaling leading to IL-6 production. Thus, we performed a quantitative comparison of lymphocyte and macrophage subsets between WT and TCRδ KO mice (see gating strategy and representative plots in Supplementary Table 2 and Supplementary Fig. 9). CD4^+^ and CD8^+^ T_conv_ cells showed a significant reduction whereas cells negative for CD4 and CD8 (double-negative (DN) T cells) showed a significant increase in TCRδ KO mice (Fig. 7f). Among the macrophage populations, M2-like (CD11c^neg^ CD206^+^) macrophages showed a significant reduction in TCRδ KO mice. On the other hand, M1-like macrophages gated as M1.1 (CD11c^+^ CD206^−^) and M1.2 (CD11c^+^ CD206^low^) were not significantly changed. DN macrophages (CD11c^neg^ CD206^neg^) showed a significant increase in TCRδ KO mice (Fig. 7g). Collectively, these data suggest that depletion of γδ T cells in aged mice attenuates several hallmarks of aging [38], including metabolic dysfunction and inflammation, which result from an interplay between γδ T cells and non-immune adipose stromal cells.

## Discussion

Despite adipose tissue dysfunction being a common underlying phenotype in both aging and obesity, clear differences in the cellular composition contributing to these altered states have been uncovered [39]. Of note are the changes in adipose tissue macrophages (ATMs) and regulatory T cells (T_regs_). ATMs have been well-studied in obesity and reports indicate a tremendous increase in the obese state (5–10% of SVF in lean state vs. 40–50% of SVF in obese) concomitant with a shift toward metabolic activation and a proinflammatory phenotype [39–41]. Contrarily, in aging, ATM numbers are not changed or are even modestly decreased [42–44], although double negative ATMs (CD11c^neg^CD206^neg^) show a trend toward increasing [43]. Regulatory T cells (T_regs_), generally regarded as anti-inflammatory, decrease in obesity thus contributing to the proinflammatory state [45]; in aging, however, T_regs_ are enriched in adipose tissue and selective depletion improves glucose homeostasis [13]. These prime examples set the stage for a new field of study to elucidate similarities and differences in adipose tissue dysfunction in aging and obesity. While many immune cell subpopulations have been studied extensively in the field of obesity, the roles of these cells in aging are largely unknown. Understanding changes in these two states may aid in the identification of universal underlying contributors to metabolic disease, which could render novel therapeutic strategies [39].

The presence of γδ T cells in adipose tissue, along with diet-induced alterations in abundance, has been previously reported [24–27, 46]. γδ T cells in VAT of lean young mice were recently shown to contribute to homeostasis via regulation of thermogenesis [27]. In their study, Kohlgruber et al. highlight that the PLZF^+^IL-17A-producing γδ T cell subset is specifically responsible for the effects on thermogenesis by regulating adipose T_reg_ accumulation in young adult mice [27]. A prior report also highlighted that a majority of adipose tissue γδ T cells produce IL-17A, and that adipose tissue IL-17A is derived primarily from γδ T cells [25]. Experiments using parabiosis and intravascular labeling have shown that adipose tissue γδ T cells in young mice are almost entirely tissue-resident [27, 46]; this is unique given that other organs show recirculation of γδ T cells. Whole mount imaging of VAT further showed that γδ T cells are dispersed throughout the tissue and not restricted to specific neural or lymphoid niches, like other T cells [46]. Using different models of diet-induced obesity (DIO), three studies have shown an increase in adipose tissue γδ T cells [24–26]. Caspar-Bauguil et al. and Zuniga et al. reported a significant increase in γδ T cells in inguinal SAT of obese mice, but not in VAT [24, 25], while Mehta et al. reported that γδ T cells significantly increased in VAT of obese mice [26]. These contradicting findings are potentially due to differences in diet composition and duration. Mehta et al. linked accumulating γδ T cells to obesity-associated inflammation and insulin resistance by showing that TCRδ KO mice were protected from these diet-induced pathologies [26]. Zuniga et al. showed no difference in HFD-induced insulin resistance between wild-type and TCRδ KO mice [25]. As the study by Zuniga and colleagues did not identify an increase in VAT γδT cells, the collective findings might indicate that γδ T cells specifically in VAT contribute to inflammation and insulin resistance in the obese state.

Our study shows that similar to obesity, γδ T cells increase in VAT by aging; the magnitude of the increase being more dramatic in aged mice compared to HFD-fed young mice. Additionally, while HFD increased γδ T cell number in VAT in proportion to an increase in fat mass, aging resulted in increased VAT γδ T cells irrespective of fat mass. HFD, in combination with aging, further increased γδ T cell number suggesting an additive effect. Similar to prior literature [25, 27, 46], we found that the majority of VAT γδ T cells are IL-17A-producing cells and express a tissue-resident memory phenotype. The degree of tissue residency was not affected by aging with at least 80% of γδ T cells in both young and aged VAT expressing the phenotype. However, both percentage and total number of IL-17A^+^ γδ T cells increased significantly by aging. As IL-17A^+^ γδ T cells are known to promote inflammation in various infectious and inflammatory diseases, it is natural to expect that an age-associated increase in IL-17A^+^ γδ T cells in VAT could promote chronic adipose tissue inflammation in aging.

Using a transcriptomics approach, we further characterized VAT γδ T cells and their age-associated differences. First, we found that the overall transcriptome of circulating γδ T cells is distinct from tissue-resident VAT γδ T cells. We also showed that within the VAT, conventional αβ T cells (T_conv_) are distinct from tissue-resident γδ T cells. While T_conv_ were enriched with transcripts characteristic of adaptive T cells, γδ T cells displayed a phenotype resembling innate myeloid cells with high representation of genes related to inflammation. Among VAT γδ T cells, we found several genes displaying age-related alterations in expression, which were predominantly associated with inflammation, immune cell composition, and adipocyte differentiation. A few of interest are discussed here along with potential functional significance. Complement component 6, which increased 13.4-fold in aged γδ T cells, was recently reported to facilitate NLRP3 inflammasome activation [47]. Thus, increased C6 in aged VAT γδ T cells could be linked to the inflammatory role of these cells. Complement activation in adipose tissue has also been implicated in low grade chronic inflammation associated with obesity [48]. Cxcl13 was increased 9.6-fold in aged VAT γδ T cells. As this chemokine is selectively chemotactic for B cells, a potential link between increased γδ T cells and increased B cells in aged VAT can be inferred. Interleukin 5 receptor alpha subunit (IL-5rα), which increased 9.1-fold in aged VAT γδ T cells, is the ligand specific subunit of the IL-5 receptor, which binds IL-5 and initiates downstream signaling. The most notable roles of IL-5 include modulating allergic and eosinophilic inflammatory diseases and regulating B1 cell abundance [49]. However, IL-5 has also been implicated in the regulation of immune responses in adipose tissue [50]. Within AT, IL-5 is largely produced by group 2 innate lymphoid cells (ILC2), which are responsible for accumulation of VAT eosinophils and alternatively activated macrophages [51]. Thus, the increase in expression of IL-5rα on γδ T cells may hint at an important crosstalk between IL-5-producing ILC2s and IL-5rα^+^γδ T cells. Thrombospondin 1 and complement factor D have been implicated as drivers of preadipocyte proliferation and differentiation [52, 53]; thus their age-related loss of expression in γδ T cells may suggest a link between γδ T cells and the known decrease in adipogenesis with age [6]. Likewise, as IL-17A has been shown to inhibit adipogenesis in vitro [25], the increased abundance of IL-17A^+^ γδ T cells in aged VAT may support their potential role in the loss of adipogenic potential with age.

Our study using indirect calorimetry to assess metabolic fitness showed a higher respiratory exchange ratio (RER) in aged mice lacking γδ T cells compared to age-matched WT mice. Generally, lower RER in aged mice compared to young mice has been portrayed as a marker of age-associated metabolic dysfunction [13, 34]. Thus, increased RER in aged mice lacking γδ T cells is suggestive of improved metabolic fitness. The concomitant increase in energy expenditure (EE) in these mice is intriguing. Brown adipose tissue (BAT) activity, including the appearance of brown/beige adipocytes within VAT, increases energy expenditure and is thought to contribute to metabolic fitness [54–58]. Aging decreases BAT abundance and VAT browning, contributing to reduced metabolic health in old age [57, 58]. Unpublished data from our lab show a sixfold increase in UCP-1 gene expression, a marker of adipocyte browning, in VAT of aged TCRδ KO mice compared to age-matched WT mice. This finding suggests that increased VAT browning in the absence of γδ T cells may contribute to increased EE, RER, and metabolic fitness in TCRδ KO mice.

We also found that aged mice lacking γδ T cells have reduced levels of IL-6 in VAT and plasma. This is suggestive of a more youthful phenotype given that increased IL-6 in aging has long been associated with a decline in physical function, heightened inflammation, senescence, and mortality [7, 59–61]. While IL-6 is but one cytokine, its importance was highlighted in a recent report showing that IL-6 can single-handedly drive features of frailty in mice [62]. The cellular source of IL-6 has also been shown to affect phenotype and downstream signaling. For example, in obesity, adipocyte-derived IL-6 promotes macrophage infiltration while myeloid-derived IL-6 suppresses it [63]. In the current study, we show that preadipocytes and ADSCs (CD45^neg^CD31^neg^ cells) are the major IL-6 producing cells in VAT of aged mice, and that a significant reduction of IL-6^+^ cells in this subset is responsible for the overall reduction of IL-6 in aged mice lacking γδ T cells. This suggests that γδ T cells, either by direct communication or via signaling with other immune cells, mediate IL-6 production by preadipocytes/ADSCs. IL-17A, which is almost exclusively produced by γδ T cells in VAT [25], has been shown to induce the expression of IL-6 in cultured preadipocytes [25] and fibroblasts [64], supporting the premise that γδ T cells may directly induce preadipocyte IL-6 production via IL-17A signaling. Alternatively, changes in the abundance of other immune cell populations mediated by the loss of γδ T cells may facilitate the overall decrease in inflammation. For example, CD4^+^ and CD8^+^ T_conv_ cells are increased in adipose tissue with age and promote inflammation [43, 65, 66]. We show that these cells are reduced in aged TCRδ KO mice, thus potentially lessening age-related T_conv_cell-mediated inflammation.

Another mechanism in the adipose tissue known to promote inflammation is senescence [6, 7]. Senescent preadipocytes are known to accumulate in adipose tissue with age and secrete a wide array of inflammatory mediators in detriment of metabolic homeostasis [6, 7]. In fact, senescent preadipocytes are thought to be the most abundant type of senescent cell in the body [6, 67]. While we did not directly evaluate senescence in this study, IL-6 is often utilized as a marker of senescence. Our data show that IL-6 is specifically decreased in the preadipocyte fraction of TCRδ KO mice. This may suggest a decrease in the proportion of senescent to non-senescent preadipocytes in VAT and raises the hypothesis that γδ T cells contribute to preadipocyte senescence.

In summary, we describe an age-associated increase in IL-17A-producing, adipose tissue-resident γδ T cells which influence adipose tissue inflammation and metabolic health. In addition to the quantitative increase, VAT γδ T cells show age-related differences in expression of genes related to inflammation, immune cell composition, and adipocyte differentiation. The accumulation of γδ T cells in VAT contributes to age-associated chronic inflammation by increasing IL-6 production from resident non-immune stromal cells, primarily preadipocytes and ADSCs. The selective decrease in VAT IL-6 is also reflected in the plasma, suggesting both a local and systemic influence of VAT γδ T cells on inflammation. This work provides insight into the complex interaction between adipose-resident immune cells and stromal cells, and implicates γδ T cells as mediators of age-related adipose tissue dysfunction.

## Supplementary Information

Below is the link to the electronic supplementary material.

Supplementary file1 (PDF 865 KB)

Supplementary file2 (PDF 129 KB)

Supplementary file3 (PDF 122 KB)

Supplementary file4 (PDF 177 KB)
