# Supplementary material for: Accumulation of γδ T cells in visceral fat with aging promotes chronic inflammation
Source: GeroScience. 2022 Apr 28;44(3):1761–78. doi: 10.1007/s11357-022-00572-w (PMC9213615; doi:10.1007/s11357-022-00572-w)
Supplement: Supplementary file 1 — Supplementary file1 (PDF 865 KB) [file 11357_2022_572_MOESM1_ESM.pdf]

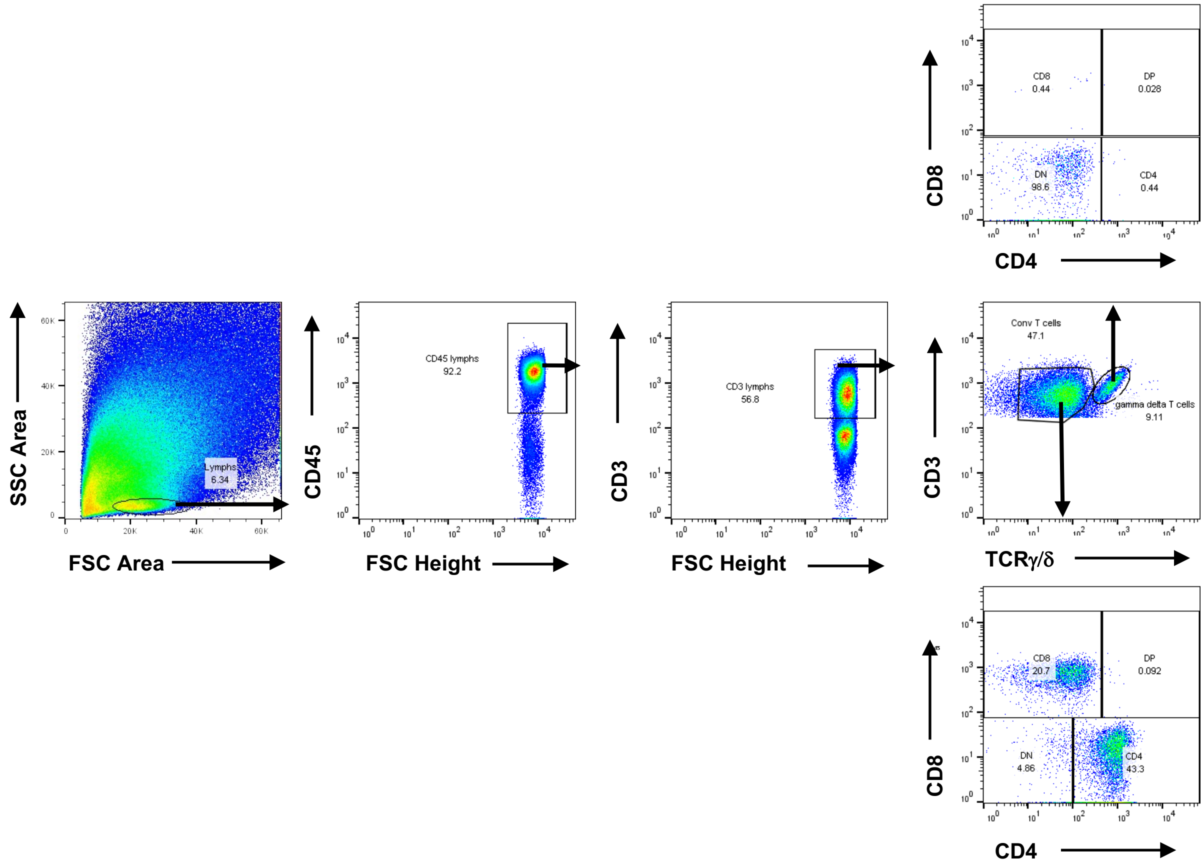

Supplementary Figure 1. Gating scheme for Figure 1. Representative flow cytometry plots to identify γδ T cells.

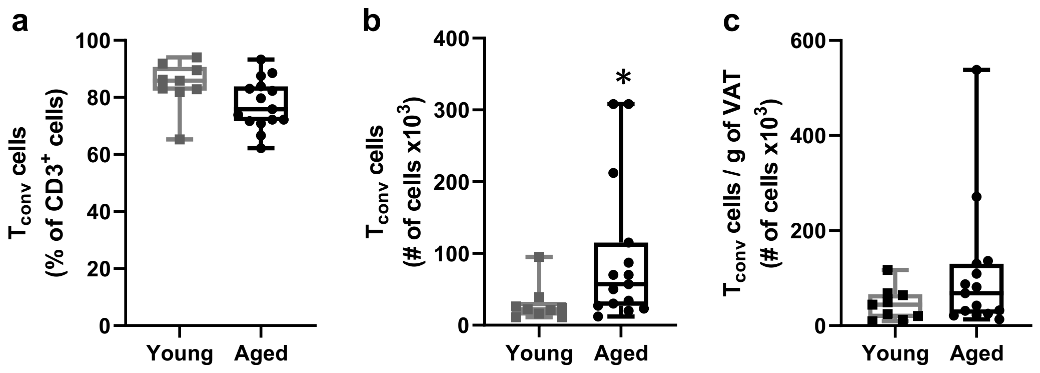

Supplementary Figure 2. Changes in VAT T_{conv} cells by aging. (a) Percentage, **(b)** Total number, and **(c)** Number per gram of adipose tissue of T_{conv} cells was quantified in young (4-6 months, n=9) and aged (19-25 months, n=15) male mice. Statistical differences were determined by Student's t-test. * $p < 0.05$.

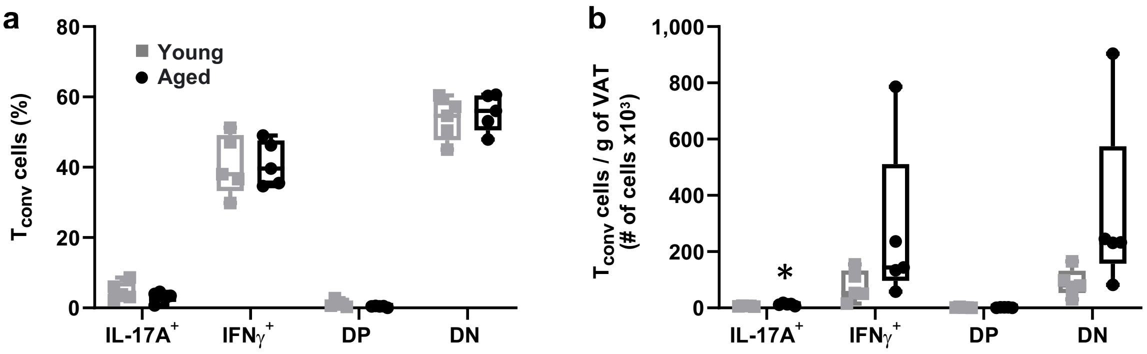

Supplementary Figure 3. IL-17A and IFNγ in VAT T_{conv} cells. T_{conv} cells from visceral adipose tissue of young (7 months, n=5) and aged (23-24 months, n=5) mice were assessed for intracellular IL-17A and IFNγ expression. **(a)** Percentage of total T cells and **(b)** Number of T_{conv} cells per gram of adipose tissue for each intracellular stain. Data are expressed in box plots from minimum to maximum values with a bar representing the mean; each symbol represents an individual mouse. Statistical differences were determined by Student's t-test. * $p < 0.05$.

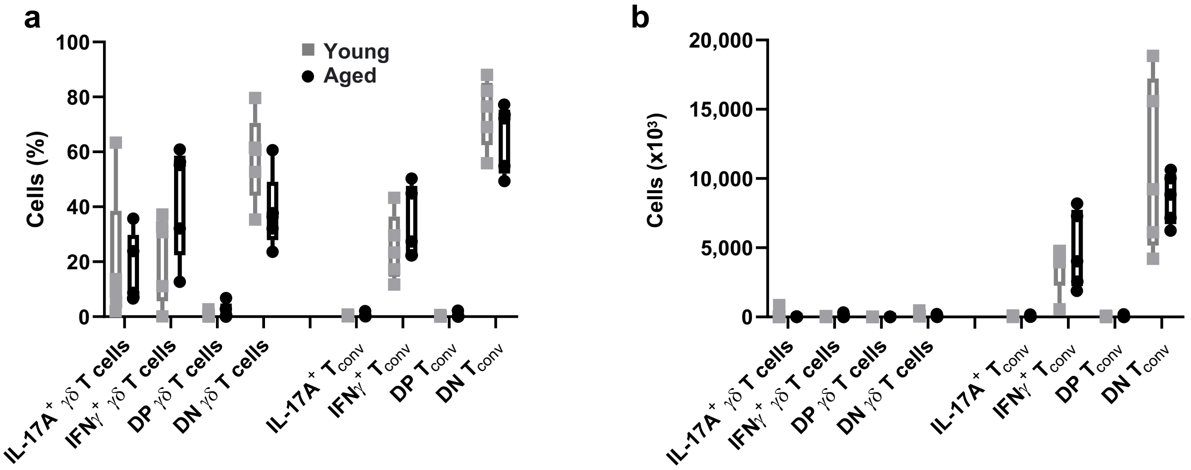

Supplementary Figure 4. IL-17A and IFNγ in splenic T cells. γδ T cells and T_{conv} cells from spleen of young (7 months) and aged (23-24 months) mice were assessed for intracellular IL-17A and IFNγ expression. **(a)** Percentage and **(b)** Total number of T cells for each intracellular stain. Data are expressed in box plots from minimum to maximum values with a bar representing the mean; each symbol represents an individual mouse. Statistical differences were determined by Student's t-test.

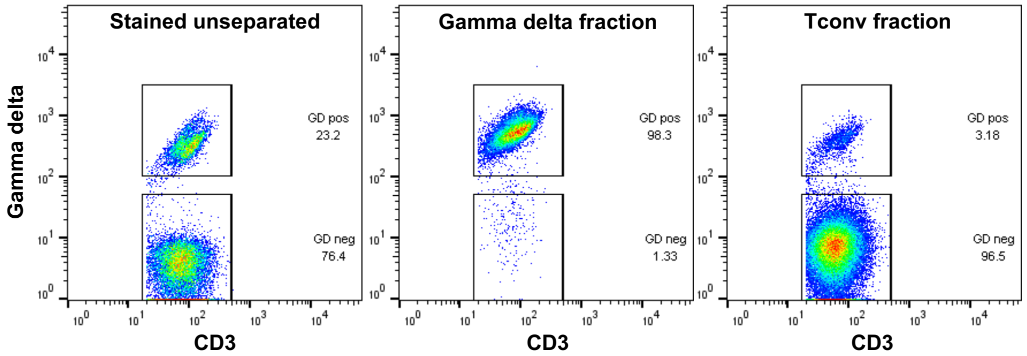

Supplementary Figure 5. Validation of magnetic separation method to obtain purified γδ T cells and T_{conv} cells. After immunomagnetic purification, cells were subjected to flow cytometry to assess purity of each fraction.

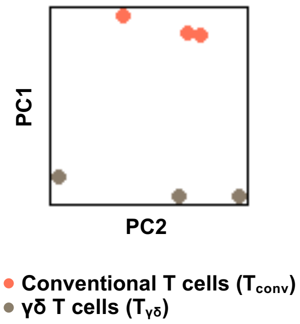

Supplementary Figure 6. Principal component analysis of the gene expression data plotted against each other for VAT γδ T cells vs. VAT T_{conv} cells.

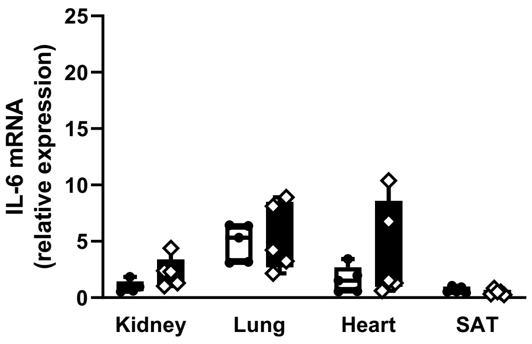

Supplementary Figure 7. IL-6 expression in tissues of WT vs. TCRδ KO mice. IL-6 gene expression in kidney, lung, heart, and subcutaneous adipose tissue (SAT) of aged WT and aged TCRδ KO mice (24-27 months, n=5) was measured by qRT-PCR.

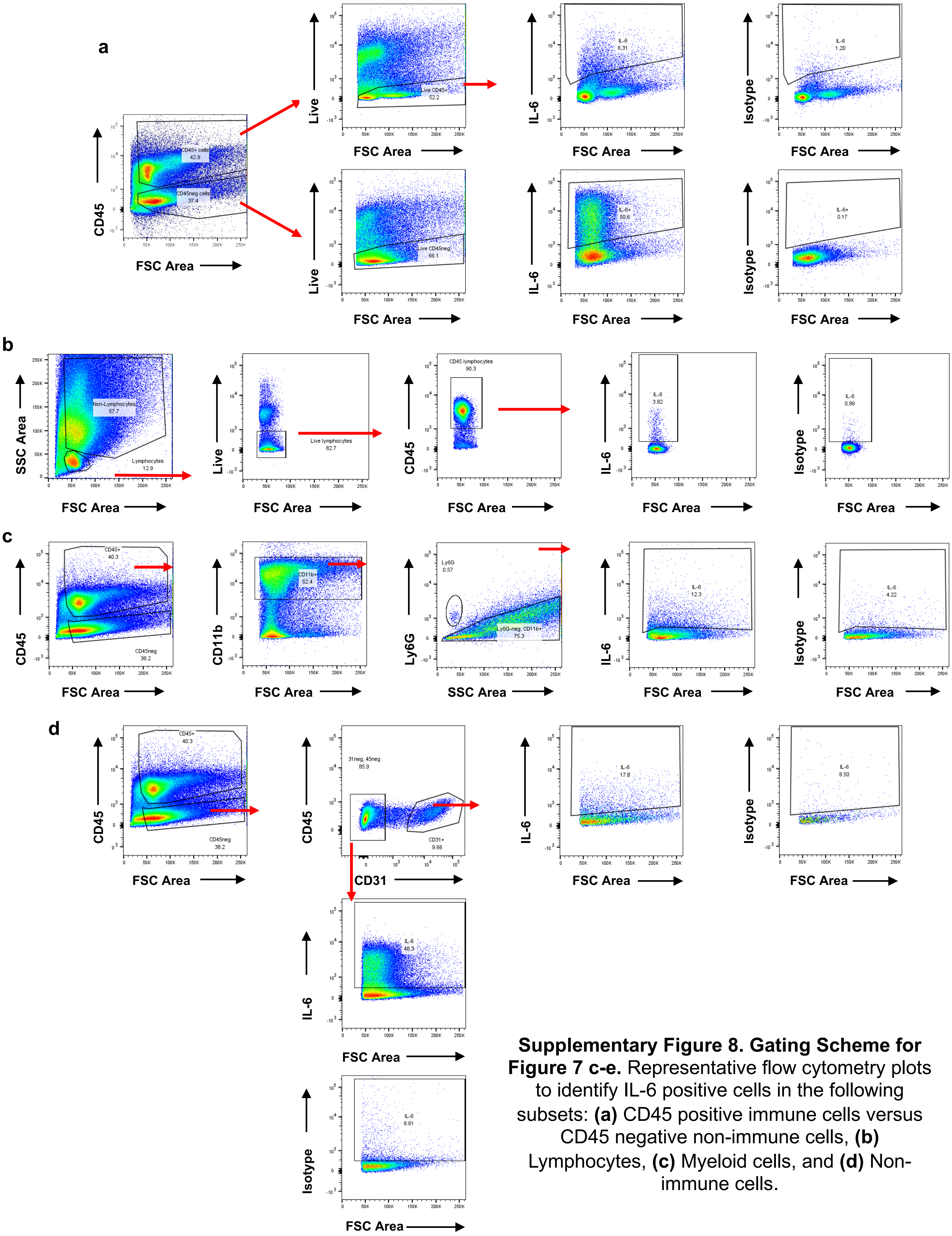

Supplementary Figure 8. Gating Scheme for Figure 7 c-e. Representative flow cytometry plots to identify IL-6 positive cells in the following subsets: **(a)** CD45 positive immune cells versus CD45 negative non-immune cells, **(b)** Lymphocytes, **(c)** Myeloid cells, and **(d)** Non-immune cells.

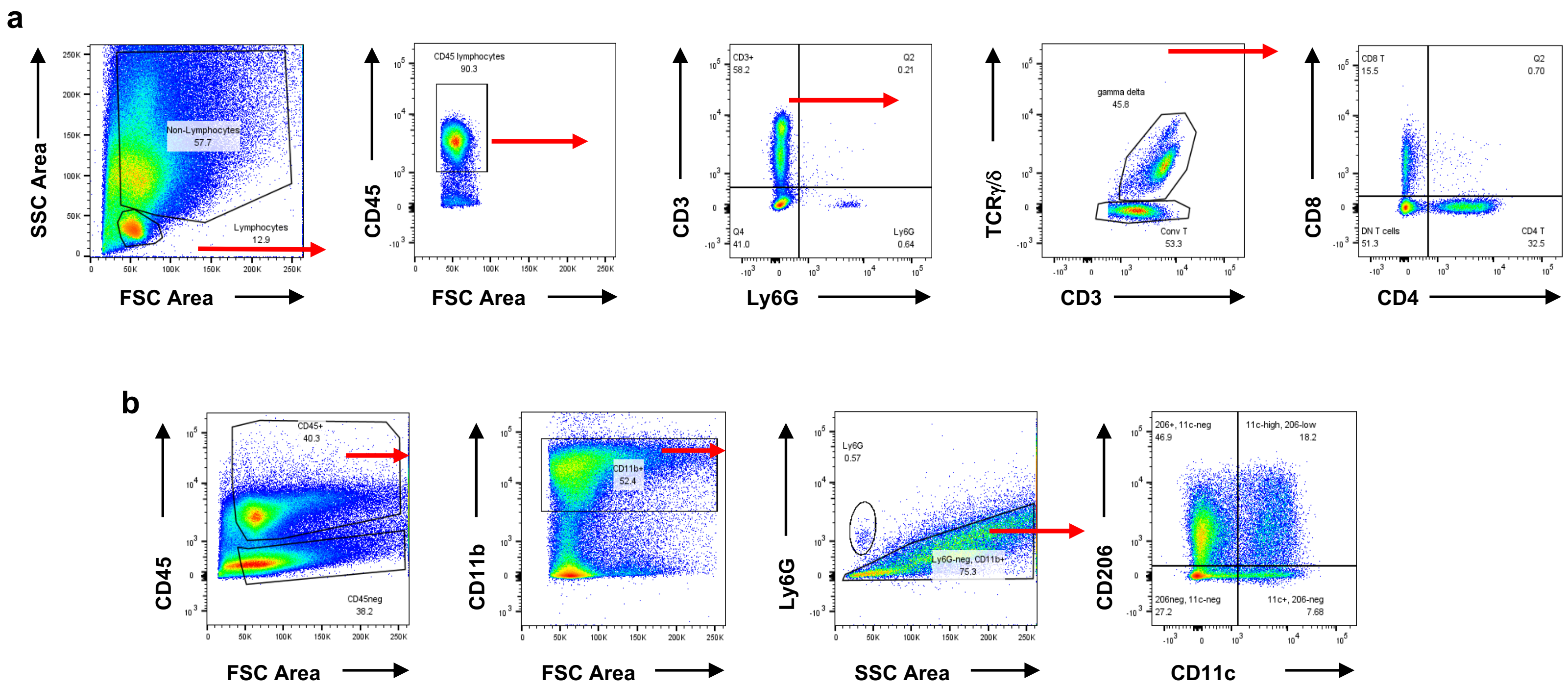

Supplementary Figure 9. Gating Scheme for Figure 7 f-g. Representative flow cytometry plots to identify **(a)** lymphocyte and **(b)** myeloid cell subsets.
