# Supplementary material for: Accumulation of γδ T cells in visceral fat with aging promotes chronic inflammation
Source: GeroScience. 2022 Apr 28;44(3):1761–78. doi: 10.1007/s11357-022-00572-w (PMC9213615; doi:10.1007/s11357-022-00572-w)
Supplement: Supplementary file 2 — Supplementary file2 (PDF 129 KB) [file 11357_2022_572_MOESM2_ESM.pdf]

Supplementary Table 1. Antibodies used in this study

Antigen	Conjugate	Manufacturer	Identifier
**Mouse**			
CD45	APC/Cyanine7	BioLegend	103116
	Brilliant Violet 510	BioLegend	103138
CD3	FITC	BioLegend	100204
TCRγ/δ	APC-Vio 770	Miltenyi Biotec	130-126-042
	PerCP-Vio 700	Miltenyi Biotec	130-117-665
	PerCP/Cy5.5	BioLegend	118118
CD11b	APC	BioLegend	101212
Ly6G	PerCP/Cyanine5.5	BioLegend	127616
CD4	PE/Cyanine7	BioLegend	100422
CD8α	Brilliant Violet 711	BD Biosciences	563046
CD11c	APC/Cyanine7	BioLegend	117324
CD206	Brilliant Violet 711	BioLegend	141727
CD34	Brilliant Violet 421	BioLegend	152207
CD31	PE/Cyanine7	BioLegend	102417
IL-6	PE	BioLegend	504503
IgG1, κ (Isotype for IL-6)	PE	BioLegend	400408
CD44	PE	BioLegend	103008
CD62L	Brilliant Violet 421	BioLegend	104436
CD69	PE-CF594	BD Biosciences	562455
IL-17A	Brilliant Violet 605	BioLegend	506927
IgG1, κ (Isotype for IL-17A)	Brilliant Violet 605	BioLegend	400433
IFN-γ	Alexa Fluor® 700	BioLegend	505823
IgG1, κ (Isotype for IFN-γ)	Alexa Fluor® 700	BioLegend	400420
**Human**			
CD45	APC	Invitrogen	17-0459-42
CD3	PE/Cyanine7	Invitrogen	25-0036-42
TCRγ/δ	FITC	Invitrogen	11-9959-42
