# Supplementary material for: Accumulation of γδ T cells in visceral fat with aging promotes chronic inflammation
Source: GeroScience. 2022 Apr 28;44(3):1761–78. doi: 10.1007/s11357-022-00572-w (PMC9213615; doi:10.1007/s11357-022-00572-w)
Supplement: Supplementary file 3 — Supplementary file3 (PDF 122 KB) [file 11357_2022_572_MOESM3_ESM.pdf]

Supplementary Table 2. Cell Identification Schemes

Cell type	Identification Scheme
Immune	$CD45^{+}$
Non-Immune	$CD45^{neg}$
Lymphocytes (total)	Lymphocyte gate
γδ T cells	Lymphocyte gate, $CD45^{+}$, $CD3^{+}$, $TCR\gamma\delta^{+}$
T_{conv} cells	Lymphocyte gate, $CD45^{+}$, $CD3^{+}$, $TCR\gamma\delta^{neg}$
CD4+ T_{conv} cell	Lymphocyte gate, $CD45^{+}$, $CD3^{+}$, $TCR\gamma\delta^{neg}$, $CD4^{+}$
CD8+ T_{conv} cell	Lymphocyte gate, $CD45^{+}$, $CD3^{+}$, $TCR\gamma\delta^{neg}$, $CD8\alpha^{+}$
DN (Double-Negative) T^{conv} cell	Lymphocyte gate, $CD45^{+}$, $CD3^{+}$, $TCR\gamma\delta^{neg}$, $CD4^{neg}$, $CD8\alpha^{neg}$
Naïve T cell	Lymphocyte gate, $CD45^{+}$, $CD3^{+}$, $CD44^{low}$, $CD62L^{hi}$, $CD69^{neg}$
Central memory T cell	Lymphocyte gate, $CD45^{+}$, $CD3^{+}$, $CD44^{hi}$, $CD62L^{hi}$, $CD69^{neg}$
Effector memory T cell	Lymphocyte gate, $CD45^{+}$, $CD3^{+}$, $CD44^{hi}$, $CD62L^{low}$, $CD69^{neg}$
Tissue-resident T cell	Lymphocyte gate, $CD45^{+}$, $CD3^{+}$, $CD44^{hi}$, $CD62L^{low}$, $CD69^{+}$
Macrophages (total)	$CD45^{+}$, $CD11b^{+}$, $Ly6G^{neg}$
M1 macrophages	$CD45^{+}$, $CD11b^{+}$, $Ly6G^{neg}$, $CD11c^{+}$, $CD206^{neg}$ or $CD206^{low}$
M2 macrophages	$CD45^{+}$, $CD11b^{+}$, $Ly6G^{neg}$, $CD11c^{neg}$, $CD206^{+}$
DN (Double-Negative) macrophages	$CD45^{+}$, $CD11b^{+}$, $Ly6G^{neg}$, $CD11c^{neg}$, $CD206^{neg}$
Neutrophils	$CD45^{+}$, $CD11b^{+}$, $Ly6G^{+}$
Endothelial cells	$CD45^{neg}$, $CD31^{+}$
Preadipocytes and adipose-derived stem cells (ADSC)	$CD45^{neg}$, $CD31^{neg}$
