# Supplementary material for: Accumulation of γδ T cells in visceral fat with aging promotes chronic inflammation
Source: GeroScience. 2022 Apr 28;44(3):1761–78. doi: 10.1007/s11357-022-00572-w (PMC9213615; doi:10.1007/s11357-022-00572-w)
Supplement: Supplementary file 4 — Supplementary file4 (PDF 177 KB) [file 11357_2022_572_MOESM4_ESM.pdf]

Supplementary Table 3. Genes differentially expressed between $\gamma\delta$ T and T_{conv} cells in aged VAT.

Gene Symbol	Log2 fold change	Linear fold change	p-value	Pathway
**Upregulated in gamma delta T cells**				
Siglec1	5.1	33.4	0.0001	Adhesion, CD molecules, Transporter Functions
Tlr5	4.4	20.5	0.0007	Inflammation, Innate, Interleukins, TLR
C6	4.3	19.3	0.001	Apoptosis, Complement Pathway, Innate
Lyve1	4.3	19.2	0.0009	Adhesion, Transporter Functions
Ccl8	4.2	18.7	0.0001	Adaptive, Cancer Progression, Chemokines & Receptors, Cytokines & Receptors, Inflammation
Tnfsf13	4.2	18.3	0.002	CD molecules, Cytokines & Receptors, TNF Superfamily
C1qa	4.2	17.7	0.0001	Complement Pathway, Innate
C1qb	4.0	16.4	0.0001	Complement Pathway, Innate
F13a1	4.0	16.3	0.0007	Basic Cell Functions
Pdgfc	4.0	15.9	0.002	Basic Cell Functions
Ccl12	3.9	14.9	0.001	Adaptive, Chemokines & Receptors, Cytokines & Receptors, Humoral, Inflammation
Cd33	3.8	14.1	0.0006	Adhesion, CD molecules
Mrc1	3.8	14.0	0.0005	CD molecules
C4b	3.8	13.9	0.0005	Complement Pathway, Humoral, Inflammation, Innate
Cxcl13	3.8	13.6	0.0001	Adaptive, B-Cell Functions, Chemokines & Receptors, Cytokines & Receptors, Humoral, Inflammation, T-Cell Functions
Apoe	3.6	12.1	0.0002	Apoptosis, Cancer Progression, Transporter Functions
Ccl24	3.6	11.9	0.0008	Adaptive, Chemokines & Receptors, Cytokines & Receptors, Inflammation
Ccl7	3.6	11.8	0.002	Adaptive, Cancer Progression, Chemokines & Receptors, Cytokines & Receptors, Humoral, Inflammation, NK Cell Functions, T-Cell Functions
Fcgr3	3.5	11.1	0.0001	Antigen Processing, CD molecules, MHC, Transporter Functions
Clec4n	3.5	11.1	0.001	Cytokines & Receptors, Innate
C3ar1	3.5	10.9	0.0002	Adaptive, Cancer Progression, Complement Pathway, Inflammation, Macrophage Functions
Trem2	3.4	10.9	0.0001	Basic Cell Functions, Humoral
Cd163	3.4	10.8	0.001	Cancer Progression, CD molecules, Inflammation
Emr1	3.3	10.0	0.00004	Basic Cell Functions
Cfh	3.3	9.86	0.001	Complement Pathway, Innate
Csf1r	3.3	9.66	0.0001	CD molecules, Chemokines & Receptors, Cytokines & Receptors, Innate, Macrophage Functions
C5ar1	3.3	9.53	0.0006	Chemokines & Receptors, Complement Pathway, Humoral
Ccl6	3.2	9.41	0.00002	Chemokines & Receptors, Cytokines & Receptors
Clec5a	3.2	9.39	0.001	Apoptosis, Cytokines & Receptors, Innate
Tlr8	3.2	9.33	0.0004	CD molecules, Inflammation, Innate, Interleukins, Pathogen Response, TLR
Gbp2b	3.2	9.24	0.005	Basic Cell Functions
Fcgr1	3.1	8.51	0.0001	Antigen Processing, CD molecules, Innate, MHC, Transporter Functions
Oas2	3.1	8.49	0.002	Basic Cell Functions
Cd63	3.1	8.34	0.0004	Adhesion, CD molecules
Timd4	3.0	7.86	0.002	Basic Cell Functions

Ccl2	3.0	7.83	0.003	Chemokines & Receptors, Cytokines & Receptors, Humoral, Inflammation, Innate, Interleukins, Macrophage Functions, NK Cell Functions, Pathogen Response, T-Cell Functions
Tnfsf12	3.0	7.80	0.001	Apoptosis, Cytokines & Receptors, TNF Superfamily
Cfp	2.0	7.69	0.00003	Cancer Progression, Complement Pathway, Innate
Mertk	2.9	7.61	0.0007	Adhesion, Apoptosis, NK Cell Functions, Transporter Functions
Creb5	2.9	7.37	0.0008	Adaptive, Inflammation
Clec4a2	2.9	7.21	0.0001	Innate
Cd14	2.9	7.21	0.0002	CD molecules, Cytokines & Receptors, Inflammation, Innate, Pathogen Response, Transporter Functions
Ccl9	2.8	7.16	0.0002	Chemokines & Receptors, Cytokines & Receptors
Tlr7	2.8	7.00	0.0003	Chemokines & Receptors, Inflammation, Innate, Interleukins, Microglial Functions, Pathogen Response, TLR
Cmklr1	2.8	6.94	0.0001	Chemokines & Receptors, Interleukins, Macrophage Functions
Bst1	2.8	6.94	0.0003	CD molecules, Humoral
Lyz2	2.8	6.84	0.0001	Transporter Functions
Colec12	2.7	6.59	0.006	Innate, Transporter Functions
Msr1	2.7	6.44	0.0006	CD molecules, Macrophage Functions, Transporter Functions
Marco	2.7	6.43	0.001	Innate, Transporter Functions
Cfb	2.7	6.37	0.0006	Complement Pathway, Innate
Slc11a1	2.6	6.24	0.0001	Adaptive, Antigen Processing, Transporter Functions
Abca1	2.6	6.12	0.001	Innate, Transporter Functions
Cd68	2.6	6.08	0.00005	CD molecules
Tlr4	2.6	6.02	0.0003	Adaptive, CD molecules, Chemokines & Receptors, Inflammation, Innate, Interleukins, Pathogen Response, T-Cell Functions, TLR
Ticam2	2.6	5.93	0.0001	Cytokines & Receptors, Inflammation, Innate, Interleukins, TLR
Cd40	2.5	5.78	0.0004	Adaptive, B-Cell Functions, CD molecules, Dendritic Cell Functions, Humoral, Inflammation, T-Cell Functions, TNF Superfamily
Fcer1g	2.4	5.21	0.000003	Antigen Processing, Interleukins, MHC, Transporter Functions
Ly86	2.4	5.20	0.00003	Humoral, Inflammation, Innate, Pathogen Response
Cma1	2.4	5.18	0.002	Adaptive, Cancer Progression, Inflammation, Interleukins, T-Cell Functions
Fcgr2b	2.4	5.15	0.0005	Antigen Processing, B-Cell Functions, CD molecules, Inflammation, Interleukins, MHC, Transporter Functions
Tlr2	2.4	5.09	0.001	CD molecules, Inflammation, Innate, Leukocyte Functions, TLR
Blk	2.3	5.07	0.0003	B-Cell Functions
Itgam	2.3	5.02	0.0002	Adaptive, CD molecules, Innate, Leukocyte Functions, T-Cell Functions, Transporter Functions
Ctsh	2.3	4.89	0.0003	Apoptosis, MHC, T-Cell Functions
Tnfrsf11a	2.3	4.87	0.0001	Adaptive, CD molecules, Cytokines & Receptors, Interleukins, TNF Superfamily
Ctsl	2.3	4.85	0.003	Basic Cell Functions
Oasl1	2.3	4.80	0.03	Basic Cell Functions

Il23r	2.2	4.63	0.001	Cytokines & Receptors, Inflammation, Innate, Interleukins, Leukocyte Functions
Rorc	2.2	4.58	0.0006	Adaptive, T-Cell Functions
Ifitm2	2.2	4.57	0.0001	Innate, Interferon
App	2.2	4.45	0.0001	Apoptosis, Cell Cycle, Innate, Transporter Functions
Lamp2	2.1	4.19	0.0004	CD molecules
Ddx60	2.1	4.19	0.003	Basic Cell Functions
Csf3r	2.1	4.17	0.002	Adhesion, CD molecules, Cytokines & Receptors
Cd36	2.1	4.16	0.003	Cancer Progression, CD molecules, Innate, Transporter Functions
Ly96	2.0	4.11	0.002	Humoral, Inflammation, Innate, Pathogen Response
Lamp1	2.0	4.05	0.0002	CD molecules, Transporter Functions
Sh2d1b1	2.0	4.06	0.0184	Interferon, NK Cell Functions

**Downregulated in gamma delta T cells**				
Mapk11	-2.0	0.25	0.001	Basic Cell Functions, Innate
Ikzf2	-2.1	0.24	0.0003	T-Cell Functions
Ets1	-2.1	0.23	0.0001	Apoptosis, Cell Cycle, Senescence
Cd96	-2.1	0.23	0.03	Adhesion, CD molecules, NK Cell Functions
Btla	-2.2	0.22	0.003	B-Cell Functions, CD molecules, T-Cell Functions
Dusp4	-2.2	0.22	0.01	Basic Cell Functions, Innate
Adora2a	-2.2	0.22	0.001	Apoptosis, Inflammation
Txk	-2.2	0.22	0.0001	Adaptive, Cytokines & Receptors, Interleukins, T-Cell Functions
Tnfsf10	-2.2	0.22	0.0001	Apoptosis, CD molecules, Cytokines & Receptors, TNF Superfamily
Ccr9	-2.2	0.22	0.002	CD molecules, Chemokines & Receptors, Cytokines & Receptors, Innate
Ccr4	-2.3	0.21	0.002	Adaptive, CD molecules, Chemokines & Receptors, Inflammation, T-Cell Functions
Fyn	-2.3	0.21	0.0001	Transporter Functions
Slamf6	-2.4	0.20	0.004	CD molecules
Il10	-2.4	0.19	0.003	Adaptive, B-Cell Functions, Cytokines & Receptors, Dendritic Cell Functions, Humoral, Inflammation, Interleukins, MHC, Pathogen Response, T-Cell Functions
Il1r2	-2.4	0.19	0.02	B-Cell Functions, CD molecules, Cytokines & Receptors, Interleukins
Nt5e	-2.4	0.19	0.0002	B-Cell Functions, CD molecules, Inflammation
Tnfrsf10b	-2.4	0.19	0.003	Apoptosis, CD molecules, TNF Superfamily
Il2rb	-2.5	0.18	0.0001	CD molecules, Cytokines & Receptors, Interleukins, NK Cell Functions
Klrk1	-2.5	0.17	0.002	CD molecules, MHC, NK Cell Functions
Tcf7	-2.6	0.17	0.0002	Apoptosis, Interleukins, T-Cell Functions
Csf1	-2.6	0.17	0.0004	Cytokines & Receptors, Inflammation, Innate, Macrophage Functions, Transporter Functions
F2rl1	-2.6	0.17	0.001	Cytokines & Receptors, Inflammation, Innate, Leukocyte Functions
Tbx21	-2.7	0.15	0.001	Adaptive, T-Cell Functions
Ctla4	-2.8	0.15	0.0006	B-Cell Functions, CD molecules, T-Cell Functions
Il1rl1	-2.8	0.14	0.0005	Chemokines & Receptors, Cytokines & Receptors, Inflammation, Innate, Interleukins, Macrophage Functions, Transporter Functions

Gata3	-3.1	0.12	0.0002	Adaptive, T-Cell Functions
Tnfrsf18	-3.1	0.12	0.009	Apoptosis, TNF Superfamily
Abcb1a	-3.2	0.11	0.03	CD molecules, Cell Cycle, Transporter Functions
Cd4	-3.4	0.10	0.0003	Adaptive, B-Cell Functions, CD molecules, Innate, T-Cell Functions
H2-Q10	-3.4	0.10	0.00001	Adaptive, Antigen Processing, B-Cell Functions, MHC
Itga2	-3.4	0.10	0.001	Adhesion, B-Cell Functions, CD molecules, Leukocyte Functions
Klrd1	-3.6	0.08	0.0003	NK Cell Functions
Slamf1	-3.6	0.08	0.00001	CD molecules
Cd28	-3.6	0.08	0.0005	Adaptive, B-Cell Functions, CD molecules, Humoral, Inflammation, Interleukins, T-Cell Functions
Ccr8	-3.6	0.08	0.004	Adaptive, Chemokines & Receptors
Ctsw	-3.7	0.08	0.00005	CD molecules
Il12rb2	-3.7	0.08	0.01	Cytokines & Receptors, Interferon, T-Cell Functions
Klrb1c	-3.8	0.07	0.005	NK Cell Functions
Cd7	-4.0	0.06	0.0004	CD molecules, NK Cell Functions
Ccr7	-4.0	0.06	0.0001	Adaptive, Antigen Processing, CD molecules, Chemokines & Receptors, Humoral, Inflammation, Interferon, Interleukins, Leukocyte Functions, Macrophage Functions, T-Cell Functions
Klra6	-4.1	0.06	0.004	Adhesion, NK Cell Functions
Sell	-4.1	0.06	0.00003	Adhesion, Apoptosis, CD molecules, T-Cell Functions
Cxcr5	-4.3	0.05	0.002	B-Cell Functions, CD molecules, Chemokines & Receptors
Ccr3	-4.3	0.05	0.002	Cancer Progression, CD molecules, Chemokines & Receptors, Cytokines & Receptors, Inflammation, Innate, T-Cell Functions
Cd5	-4.3	0.05	0.000004	Apoptosis, CD molecules, T-Cell Functions
Sh2d1a	-4.4	0.05	0.0001	Humoral, NK Cell Functions
Lrrn3	-4.5	0.04	0.00002	CD molecules
Tnfsf8	-4.7	0.04	0.005	CD molecules, Cytokines & Receptors, T-Cell Functions, TNF Superfamily
Gzmm	-4.7	0.04	0.001	Innate
Cd22	-4.7	0.04	0.00002	Adhesion, B-Cell Functions, CD molecules
Cd2	-4.8	0.04	0.001	CD molecules, NK Cell Functions, T-Cell Functions
Tigit	-4.8	0.04	0.0001	Interleukins, T-Cell Functions
Il2	-4.8	0.04	0.002	Adaptive, Cytokines & Receptors, Interleukins, T-Cell Functions
Ncr1	-5.0	0.03	0.01	CD molecules
Ccl5	-5.3	0.02	0.0004	Adaptive, Cancer Progression, Chemokines & Receptors, Dendritic Cell Functions, Inflammation, Innate, Macrophage Functions, NK Cell Functions, T-Cell Functions, Transporter Functions
Xcl1	-5.4	0.02	0.0004	Chemokines & Receptors, Cytokines & Receptors, Inflammation, Innate, T-Cell Functions
Gzmk	-5.5	0.02	0.005	Innate
Gzma	-5.6	0.02	0.002	Apoptosis
Cd40lg	-5.7	0.02	0.0003	Adaptive, B-Cell Functions, CD molecules, Cytokines & Receptors, Dendritic Cell Functions, Inflammation, Interleukins, MHC, T-Cell Functions, TNF Superfamily
Cd27	-5.7	0.02	0.001	B-Cell Functions, CD molecules, T-Cell Functions, TNF Superfamily
Cd160	-5.7	0.02	0.0008	CD molecules, MHC

Foxp3	-5.8	0.02	0.0008	Adaptive, B-Cell Functions, Cytokines & Receptors, Inflammation, Interleukins, T-Cell Functions
Cd8a	-5.8	0.02	0.00002	Adaptive, CD molecules, Innate, T-Cell Functions
Eomes	-5.9	0.02	0.001	Interferon, T-Cell Functions
Tnfrsf4	-5.9	0.02	0.0002	B-Cell Functions, CD molecules, Cytokines & Receptors, Inflammation, T-Cell Functions, TNF Superfamily
Ifng	-5.9	0.02	0.0001	Adaptive, Chemokines & Receptors, Cytokines & Receptors, Humoral, Interleukins, Pathogen Response, Senescence, T-Cell Functions, Transporter Functions
Lck	-6.1	0.01	0.0002	Apoptosis, B-Cell Functions, T-Cell Functions
Cd6	-6.2	0.01	0.0001	Adhesion, CD molecules
Klrc1	-6.3	0.01	0.0002	CD molecules, NK Cell Functions
Klrg1	-6.3	0.01	0.0003	Innate
Klra7	-6.4	0.01	0.001	Adhesion, NK Cell Functions
Gzmb	-6.6	0.01	0.0009	Apoptosis, T-Cell Functions
Cd8b1	-6.8	0.01	0.0003	CD molecules, T-Cell Functions
Klrc2	-7.0	0.01	0.0007	CD molecules
Fasl	-7.0	0.01	0.001	Adaptive, Cytokines & Receptors, Inflammation, T-Cell Functions, TNF Superfamily
Prf1	-7.1	0.01	0.0000002	Apoptosis, Granzyme, Immune Response, T-Cell Functions
